# The trans-cultural geometry of liberation: convergent topologies across independent soteriological traditions Article I of III: the comparative anatomy of liberation structures

**DOI:** 10.3389/fpsyg.2026.1871395

**Published:** 2026-09-30

**Authors:** Swapan Samanta

**Affiliations:** Independent Researcher, Kolkata, India

**Keywords:** bittul ha-yesh, comparative soteriology, consciousness, cross-cultural mysticism, fanā’, Gelassenheit, liberation topology, mokṣa

## Abstract

This article demonstrates that diverse soteriological traditions across six civilisational contexts (Hindu, Buddhist, Christian mystical, Islamic Sufi, Kabbalistic Jewish, and Graeco-Roman philosophical) instantiate a shared structural grammar of liberation despite having developed their models within substantially different philosophical, linguistic, and institutional contexts. A five-element template is identified: a conditioned principle (current self), an unconditioned principle (ultimate ground), a perceived gap between them (the structural definition of bondage), a method of resolution (path), and a final stabilised state (liberation). This template resolves into exactly three topological types: closed identity (gap recognised as illusory, endpoint: stillness), open-relational (gap refined and preserved, endpoint: sustained intensity), and disentanglement (gap recognised as natural original condition, endpoint: witness-detachment). The convergence of these three types across traditions constitutes the primary evidence presented. A critical methodological finding is introduced: the Invariance Test, which demonstrates that liberation is characterised not by observable transformation but by its absence: the liberated person continues with their work as before, neither celebrating, advertising, nor restructuring identity around the event. The article refrains from offering a causal hypothesis for the convergence, reserving that analysis for Article III in this series.

## 1 Introduction: the convergence problem

Every major civilisation has produced accounts of a radical transformation in human consciousness, a passage from bondage to freedom, from confusion to clarity, from existential misalignment to resolution. These accounts vary enormously in doctrinal content, cultural context, ritual apparatus, and theological justification. Yet when we set aside the vocabulary and attend to the structure, something unexpected emerges: the same small set of formal patterns, recurring across traditions that developed their liberation models within substantially different philosophical and institutional contexts.

The convergence is not a familiar claim in comparative religion. The standard approach has oscillated between two poles. The perennialist position (Huxley, 1945; Schuon, 1984; Smith, 1976) holds that all traditions teach the same truth in different languages, achieving universality at the cost of erasing genuine structural differences. The constructivist position (Katz, 1978; Proudfoot, 1985) holds that mystical experience is shaped so thoroughly by cultural conditioning that no meaningful cross-cultural comparison is possible, achieving scholarly rigour at the cost of ignoring formal convergences that are present in the evidence. More recent comparative approaches (Hollenback, 1996; Payne, 2018; Studstill, 2005) have sought middle positions, but the tension between universality and particularity persists.

This article proposes a third approach: structural comparison without doctrinal reduction. Rather than asking whether traditions ‘teach the same thing’ (perennialism) or whether comparison is possible at all (constructivism), this study asks a more modest and more tractable question: do traditions that developed within substantially different contexts instantiate the same formal template, and if so, do they resolve that template into the same set of topological types?

### 1.1 A preliminary question: why liberation at all?

Before proceeding to the structural analysis, a prior question deserves acknowledgment. As one reviewer of an earlier version of this article observed, many religious and philosophical (including Australian Aboriginal systems, many African cosmologies, and numerous indigenous frameworks worldwide) do not emphasise liberation as a central concern. Their adherents do not experience themselves as trapped, alienated, or in need of radical transformation. The concept of a conditioned–unconditioned gap requiring resolution appears foreign to their experience.

This observation carries structural significance. The present framework predicts precisely this pattern: the three topological types identified here arise only within traditions that model human experience through a two-principle system (conditioned self, unconditioned ground). Traditions that do not model experience through such a binary structure would not produce liberation topologies, and they do not. The question of why some civilisations developed two-principle models while others maintained unified or non-binary frameworks is itself an important research question, but it exceeds the scope of the present article. What the present analysis requires is only that those traditions which do model experience through a two-principle system be compared structurally, and the evidence suggests they converge on the same three topological types.

### 1.2 A note on historical and analytical independence

This article compares traditions that developed their liberation models within substantially different philosophical, linguistic, and institutional contexts. This claim of contextual independence is deliberately distinguished from a stronger claim of absolute historical isolation, which would be unsustainable for several cases. Possible indirect pathways of intellectual transmission exist: Neoplatonic influences on Sufi thought (Chittick, 1989; Netton, 1992), possible Sufi resonances in early Hasidism (Idel, 1995), and debated questions of Buddhist influence on Pyrrho and Hellenistic thought (Beckwith, 2015). The article does not deny these possibilities.

However, the structural argument does not require absolute historical isolation. It requires only that the traditions in question developed their specific liberation models within sufficiently different conceptual, theological, and institutional environments that the convergence cannot be explained as simple borrowing of a ready-made template. Eckhart did not borrow the Advaitic template and rename it; Dōgen did not import Hasidic bittul and translate it into Zen vocabulary. Whatever indirect influences may have existed at the margins, the central liberation architectures of these traditions were developed within their own philosophical histories, in response to their own conceptual problems, using their own terminologies. That they arrived at structurally equivalent configurations is the datum requiring explanation.

Methodological note: The five-element template and three topological categories presented below were developed through an iterative process of reading primary and secondary sources across the six traditions, identifying recurring structural positions, and testing proposed categories against additional cases. The framework was not constructed in advance and then applied to the traditions; rather, the categories emerged from sustained comparative engagement and were progressively refined. The resulting classification involves interpretive judgment, as any structural reading of complex textual traditions must. The criterion for assignment to a topological type is the tradition’s treatment of the gap between conditioned and unconditioned principles: whether the gap collapses (closed), is refined and preserved (open), or is recognised as the natural condition (disentanglement). The criterion applies to specific models within traditions, not to traditions as wholes, since individual traditions may contain multiple and even competing liberation models.

## 2 The universal structural template

Across the six civilisational traditions examined in this study, five structural elements recur with sufficient regularity to constitute a shared grammar of liberation. The elements are not culturally specific content but formal positions within a template: each tradition assigns radically different content to each position while preserving the structural pattern.

### 2.1 The five elements

*Element 1: The conditioned principle*. The current identity, finite, time-bound, identified with particular form. This is the locus of bondage, the site from which the problem of liberation is experienced. It is designated variously as ātman (Advaita), nafs (Sufi), the soul or ego (Eckhart), the deluded mind (Zen), yesh (Kabbalah), or the untrained psyche (Stoic).

*Element 2: The unconditioned principle*. The ultimate ground, infinite, timeless, unconditioned by form. Designated variously as Brahman (Advaita), Allāh or al-Ḥaqq (Sufi), the Gottheit (Eckhart), Buddha-nature or śūnyatā (Zen), Ayin or Ein Sof (Kabbalah), or logos/physis (Stoic).

*Element 3: The perceived gap*. The misalignment between conditioned and unconditioned principles, which constitutes the structural definition of bondage. Whether this gap is ontological, epistemological, or phenomenological varies across traditions and constitutes a primary axis of differentiation.

*Element 4: The method of resolution*. The practice, discipline, or transformation that addresses the gap: jñāna, bhakti, or dhyāna (Hindu); zazen or kōan practice (Zen); Gelassenheit (Eckhart); fanā’ through dhikr (Sufi); bittul through prayer (Hasidic); rational self-examination (Stoic).

*Element 5: The final stabilised state*. The condition in which the tension between conditioned and unconditioned principles is resolved. The nature of this final state, particularly whether it terminates or preserves existential longing, constitutes the topological signature of the liberation model (Figure 1).

**Figure 1:**
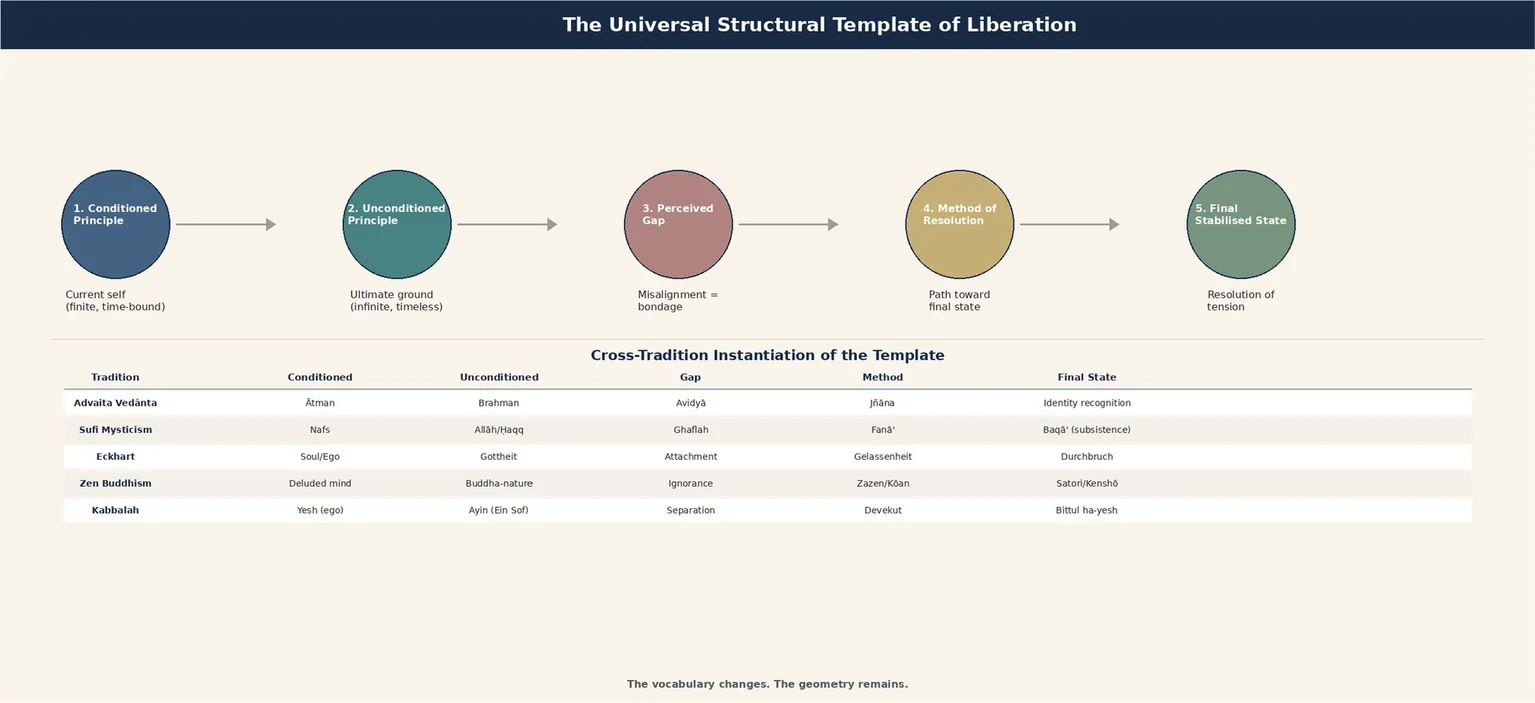
The universal five-element template with cross-tradition instantiation.

### 2.2 What the template is and what it is not

The claim advanced here is structural, not theological. The five-element template is not a claim that all traditions ‘teach the same thing’ in different vocabularies. They do not. The content assigned to each element differs radically. What Advaita calls Brahman and what Eckhart calls the Gottheit are not the same concept clothed in different words. They arise from different philosophical histories, different metaphysical commitments, different ontological frameworks.

The claim is more modest and more defensible: these different concepts occupy the same structural position within their respective liberation architectures. They are functionally equivalent without being doctrinally identical. A cardiologist and a plumber both work with ‘conduit systems’, the content differs completely, yet the structural position is the same: a system through which something essential flows. This distinction between structural equivalence and doctrinal identity is the methodological foundation of the present analysis.

The approach here is analytical rather than ontological. The five-element template is an analytical instrument for comparing liberation structures, not a claim about the ultimate nature of consciousness. Whether the identified structural convergence reflects universal properties of consciousness itself, or whether it reflects shared features of the conceptual systems that humans construct when addressing existential questions, is a further question that the present article deliberately leaves open. The companion Article III engages with this epistemological question directly.

## 3 Closed topologies across civilisations

The first topological class comprises models in which liberation culminates in the collapse of polarity between conditioned and unconditioned principles. The gap is revealed as apparent rather than real. The final state is recognition rather than achievement: the discovery that the two principles were never ontologically separate.

An important observation: traditions assigned to this topology contain substantial internal diversity. Advaita Vedānta alone encompasses multiple sub-schools (Vivaraṇa, Bhāmatī, and others) with competing interpretations of the mechanism of ignorance and the nature of the liberated state. The classification of ‘Advaita’ as closed topology refers to the structural pattern shared across these sub-schools (gap-closure through recognition), not to the specific metaphysical positions on which they disagree. The same caveat applies to each tradition discussed below.

### 3.1 Advaita Vedānta: Ātman–Brahman

Śaṅkara’s analysis of avidyā (ignorance) as adhyāsa (superimposition) (Śaṅkara, 1921) illuminates how consciousness mistakenly identifies with limited objects (body, mind, social role), just as one might mistake a rope for a snake. The jīva is not ontologically separate from Brahman but merely Brahman under the spell of beginningless ignorance. The mahāvākyas function not as metaphysical assertions to be believed but as identity-dissolving recognitions that shatter superimposed boundaries. Liberation (mokṣa) is recognition of what was always the case. The phenomenological marker: stillness, the cessation of seeking, since there is nothing to seek when the seeker is recognised as identical with the sought.

### 3.2 Meister Eckhart: Seelenfunklein–Gottheit

In Eckhart’s mysticism, the soul contains a Funklein (spark) identical in nature with the Gottheit (Godhead beyond the personal God) (Eckhart, 1994; McGinn, 2001). Liberation occurs through Durchbruch (breakthrough) and is sustained through Gelassenheit (releasement). His formulation ‘The eye with which I see God is the same eye with which God sees me’ is a precise parallel to tat tvam asi. This is not a borrowed formulation (there is no evidence of direct textual contact with Śaṅkara), but a structurally equivalent instantiation of the same closed topology developed within a fundamentally different theological and institutional context.

### 3.3 Zen Buddhism: practice–awakening

Dōgen’s Sōtō Zen (Dōgen, 2010) takes the closed topology to its most radical form: practice is not a means to awakening but its expression. The distinction between before and after realisation itself reintroduces the duality that awakening dissolves. The convergence with Advaita is structural, not doctrinal. Advaita posits a metaphysical self identical with absolute reality. Zen denies any metaphysical self whatsoever. Yet both produce the same structural outcome: gap closure through recognition, with stillness as the phenomenological marker. The doctrinal content is opposite; the topology is the same.

### 3.4 Sufi fanā’–baqā’

Fanā’ (annihilation of the ego-self in God) followed by baqā’ (subsistence) represents a two-phase closed model (Schimmel, 1975). Al-Ghazālī’s metaphor is definitive: iron placed in fire glows and behaves like fire, yet remains iron transformed by fire. The post-fanā’ state is ‘sobriety after intoxication,’ a stillness following dissolution. The possible influence of Neoplatonic thought on the conceptual vocabulary of some Sufi formulations (Netton, 1992) does not undermine the structural comparison, since the liberation architecture itself (gap-closure through ego-annihilation) was developed within an Islamic theological context fundamentally different from either Advaita or Zen.

### 3.5 Kabbalistic bittul ha-yesh

In Hasidic mysticism (Scholem, 1941), bittul ha-yesh (annihilation of somethingness) is the recognition that the ego (yesh, ‘something’) has no independent existence, that it is an apparent manifestation of Ayin (divine nothingness) (Elior, 1993; Matt, 1995). Schneur Zalman of Liadi: ‘Just as one annihilates oneself from Yesh to Ayin, so too it is drawn down from above from Ayin to Yesh.’ Some scholars have noted possible indirect Sufi influence on early Hasidism (Idel, 1995), but the structural pattern is already present in pre-Hasidic Kabbalah, and the specific formulation of bittul operates within a sefirotic framework with no parallel in Sufi metaphysics (Figure 2).

**Figure 2:**
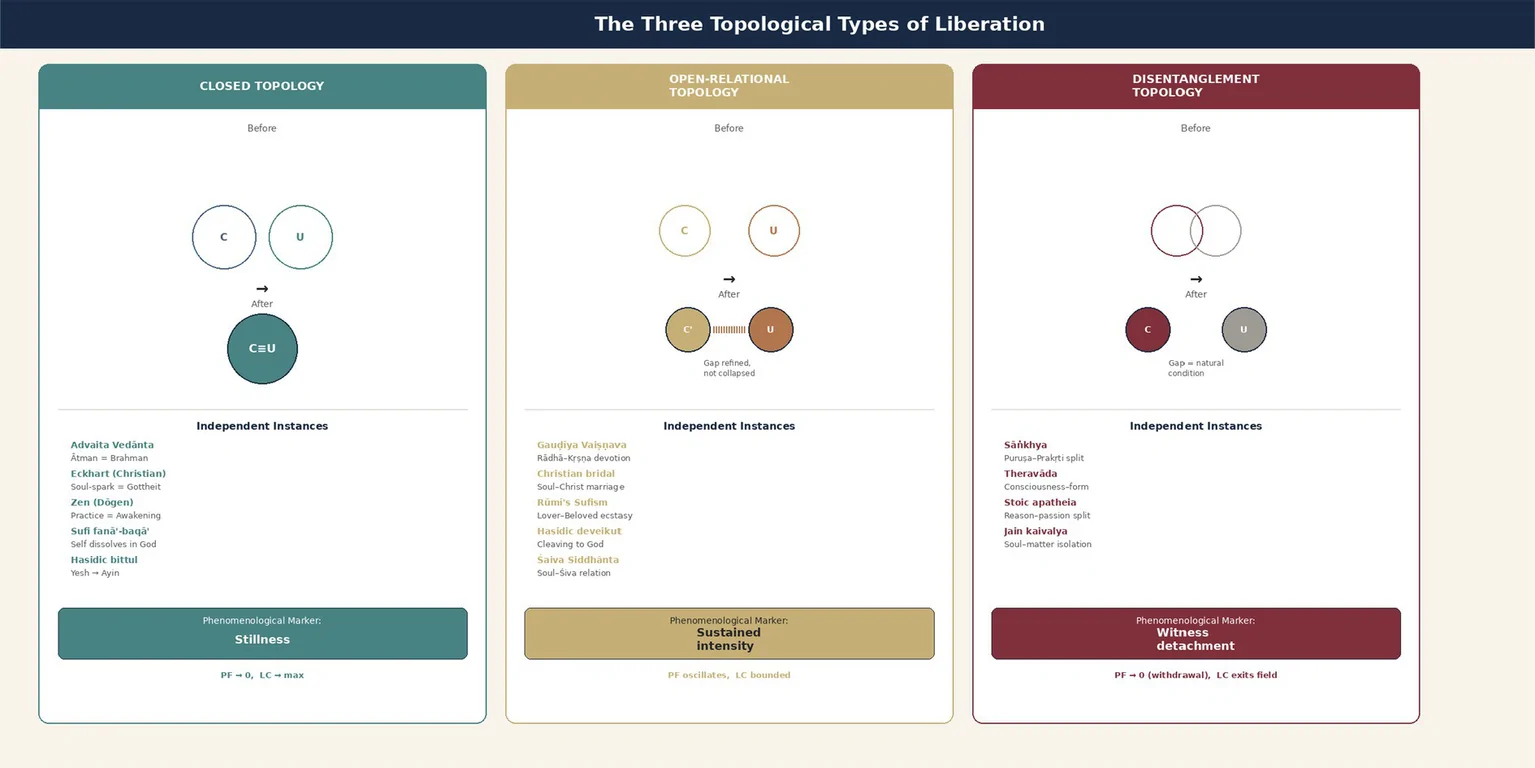
The three topological types with independent instances and phenomenological markers.

## 4 Open-relational topologies across civilisations

The second class preserves relational polarity as ultimate. The gap is not collapsed but refined, purified, and in some formulations eternalised.

### 4.1 Gauḍīya Vaiṣṇava bhakti

In Caitanya’s theology, liberation is the perfection of devotional love between the soul and God, modelled on Rādhā–Kṛṣṇa. The gap is refined into mahābhāva, the highest state of ecstatic love that requires sustained polarity. Without separation, no longing; without longing, no devotion; without devotion, no rasa.

### 4.2 Christian bridal mysticism

In Teresa of Ávila’s seventh mansion, the soul achieves permanent union yet remains a distinct entity within that union. The bride does not become the bridegroom. The gap is refined into perfect communion rather than collapsed into identity (Teresa of Ávila, 2007).

### 4.3 Rūmī’s intoxicated Sufism

Rūmī’s poetry sustains ecstatic lover-beloved intensity rather than dissolving it (Rūmī, 2004). His formulation coexists within the same tradition (Islam) as the closed fanā’ model, demonstrating that topological type is not determined by religious affiliation but by phenomenological temperament within the tradition.

### 4.4 Hasidic deveikut (non-annihilationist forms)

While Chabad Hasidism developed the annihilationist bittul ha-yesh (a closed topology), other Hasidic lineages emphasised deveikut (cleaving to God) as a relational practice in which the soul maintains its identity while adhering to the divine presence with ecstatic intensity. The Baal Shem Tov’s populist orientation, finding divine sparks in everyday life through joy, story, and communal prayer, preserves the gap between the finite self and the infinite God as the very medium of devotional engagement. This coexistence of closed and open topologies within a single tradition is among the strongest pieces of evidence that topological type is determined not by religious affiliation but by what this article terms phenomenological temperament.

## 5 Disentanglement topologies across civilisations

The third class resolves through radical differentiation. The two principles are recognised as having always been ontologically separate.

### 5.1 Sāṅkhya: puruṣa–prakṛti

Puruṣa (pure witness-consciousness) and prakṛti (material nature) are ontologically distinct. Liberation (kaivalya or aloneness) is radical disidentification, not union, not relationship, but restoration of original separateness.

### 5.2 Theravāda Buddhism

Vipassanā practice more closely resembles disentanglement than closure: bare attention to nāma-rūpa, progressively disentangling awareness from its objects. Nibbāna is not identity-recognition but cessation of craving that binds consciousness to form.

### 5.3 Stoic apatheia

The Stoic sage differentiates what is ‘up to us’ (proairesis) from everything else. Freedom consists in complete withdrawal of identification from everything that is not moral choice. This is structurally identical to Sāṅkhya’s kaivalya: liberation through differentiation rather than union.

## 6 Convergence analysis

### 6.1 The master comparison table

Table 1 presents the complete cross-tradition comparison, demonstrating that the five-element template resolves into the same three topological types across six independent civilisational contexts.

**Table 1:** Master comparison of liberation structures across twelve independent models.

Tradition	Model	Conditioned	Unconditioned	Gap Nature	Resolution	Topology	Marker
Advaita	Ātman–Brahman	Ātman	Brahman	Epistemological	Jnāna	Closed	Stillness
Eckhart	Soul–Gottheit	Soul/Ego	Gottheit	Epistemological	Gelassenheit	Closed	Stillness
Zen	Mind–Buddha	Deluded mind	Buddha-nature	Epistemological	Zazen	Closed	Stillness
Sufi	Nafs–Allāh	Nafs	Al-Ḥaqq	Ontic-epistemic	Fanā’	Closed	Sobriety
Hasidic	Yesh–Ayin	Yesh	Ein Sof	Apparent	Bittul	Closed	Stillness
Gauḍīya	Rādhā–Kṛṣṇa	Jīva	Param.	Ontological	Bhakti	Open	Intensity
Christian	Soul–Christ	Soul	Christ	Ontological	Prayer	Open	Intensity
Rūmī	Lover–Beloved	Self	Allāh	Experiential	Whirling	Open	Ecstasy
Sāṅkhya	Puruṣa–Prak.	Puruṣa	Prakṛti	Entanglement	Viveka	Disent.	Detach.
Theravāda	Vipassanā	Consciousness	Form	Craving	Insight	Disent.	Detach.
Stoic	Proairesis	Sage	Cosmos	Structural	Reason	Disent.	Detach.

### 6.2 Observations on convergence

Three observations emerge from this comparison. First, the closed topology is the most widely instantiated, appearing independently in five traditions (Advaita, Christian mystical, Zen, Sufi, Kabbalistic). This convergence is striking because the traditions differ maximally in their doctrinal content: Advaita posits a metaphysical self identical with absolute reality; Zen denies the existence of any self whatsoever; Eckhart’s framework is Trinitarian Christian; Sufi fanā’ presupposes Qur’ānic monotheism; Kabbalistic bittul operates within the sefirotic emanation system. Yet all five produce the same structural outcome: gap closure through recognition, with stillness as the phenomenological marker.

Second, the open-relational topology appears in at least four traditions (Gauḍīya Hindu, Christian bridal, Rūmī’s Sufism, Hasidic deveikut), and in each case coexists within the same broader tradition that also produces closed models. Christianity produces both Eckhart (closed) and Teresa (open). Sufism produces both fanā’ (closed) and Rūmī (open). Hasidism produces both bittul (closed) and relational deveikut (open). This coexistence demonstrates that the topological type is not determined by religious affiliation but by a deeper variable—what this article terms phenomenological temperament.

Third, the disentanglement topology, while less widely represented (Sāṅkhya, Theravāda, Stoic), constitutes a genuinely distinct third type irreducible to either of the others. Its resolution through differentiation rather than union or refined relation has no parallel in the closed or open models. Its relative rarity may reflect the fact that most soteriological traditions arise from relational concerns (the self’s relationship to God, to the absolute, to the cosmos) rather than from the analytical project of discriminating consciousness from its objects (Figure 3).

**Figure 3:**
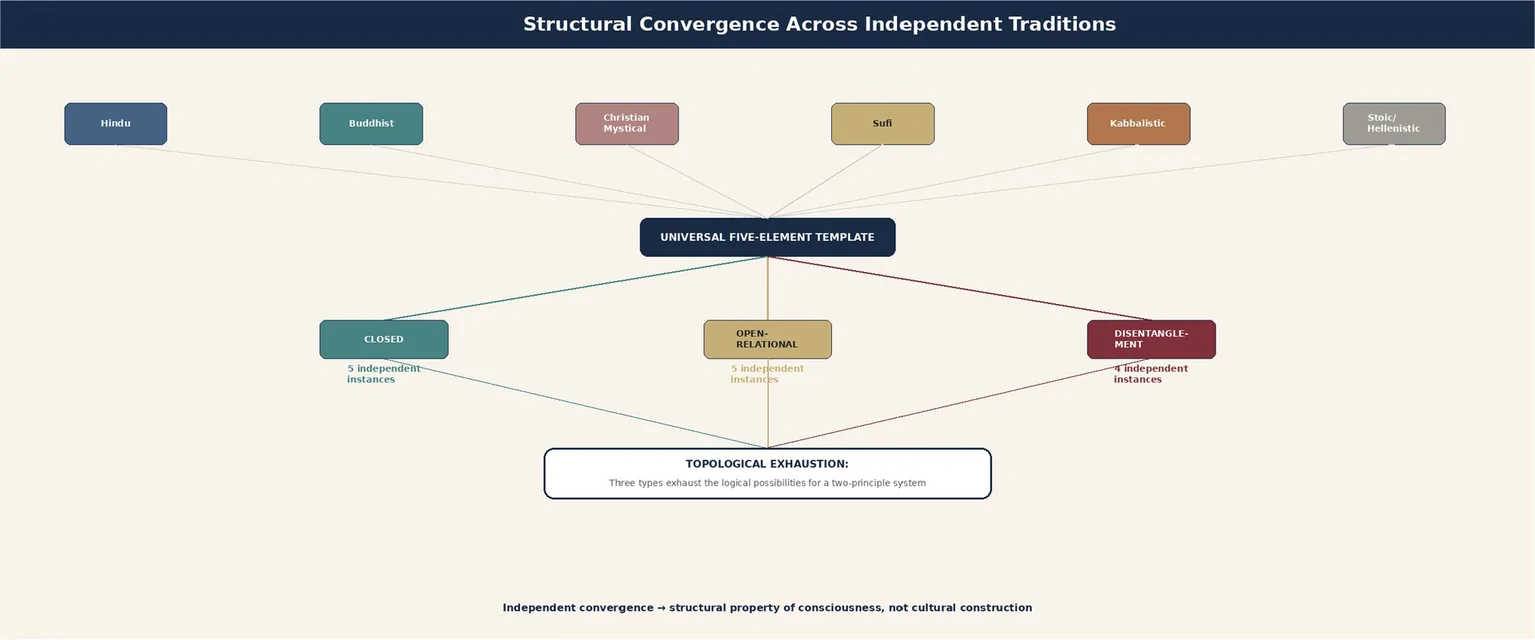
Structural convergence: six civilisational traditions resolving through the universal template into three topological types.

## 7 The invariance test: what liberation looks like from the outside

The structural analysis above identifies liberation topologies through their formal properties, specifically how they treat the gap between conditioned and unconditioned principles. But a deeper diagnostic emerges from the primary texts themselves, one that addresses the epistemological question of how liberation can be recognised at all (Samanta et al., 2026; Vālmīki, 1984–2016).

Consider two episodes from the Rāmāyaṇa. In the Ahalyā episode, Rāma’s foot touches a stone and Ahalyā ‘emerges.’ The popular reading imposes a hierarchical structure: a superior being (Rāma) liberates an inferior condition (the cursed Ahalyā). But Vālmīki’s text does not state this. Rāma does not claim the act. The text describes contact and emergence, not transaction and transformation. The hierarchical reading is a product of later commentarial reception, shaped by the very position-fixation (PF > 0 in the language of Liberation Mathematics; Samanta, 2026a) that the text itself dissolves.

Similarly, Śabarī, a tribal woman, offers Rāma half-eaten berries. The standard reading frames this through social hierarchy: a gracious deity condescends to accept polluted food from a low-caste woman. But this reading imports precisely the categories (caste, pollution, condescension) that the text does not contain. Vālmīki describes a woman who tastes each berry to ensure only the sweetest reach Rāma. The act has no position in it. The act is not performed humility, not demonstrated devotion, not a bid for liberation. She is doing what she does, with zero self-consciousness and zero performance.

What is structurally diagnostic is what happens afterward. Ahalyā does not start teaching. She does not announce her liberation. She does not gather disciples or found a sect. She returns to her life. Śabarī does not use the encounter into spiritual authority. She never becomes ‘the woman Rāma blessed.’ The text does not record either figure doing anything different after the encounter. They continued with their work, as before.

*The Invariance Test: If an encounter or realisation produces observable behavioural change in the direction of celebration, advertisement, authority-claiming, sect-founding, title-adoption, or identity-restructuring around the event, then PF > 0 and the event was not liberation. If the person continues exactly as before, doing what they were already doing, perhaps with slightly more presence and slightly less self-consciousness, then PF = 0 and the condition is consistent with liberation.*

This test has several implications. First, it resolves the epistemological question raised by structuralist approaches: the topological geometry presented in this article is not an analytical construction imposed by the researcher, because an imposed construction would itself be a position (PF > 0) that produces visible change (a new framework, a new taxonomy, a new claim). The geometry is what becomes visible when position-fixation is reduced, when the text is read not as a hierarchical narrative but as a description of contact between consciousnesses, neither of which occupies a fixed position.

Second, the Invariance Test explains why the liberation event is invisible to social observation. The commentarial tradition sees Ahalyā’s episode as a dramatic miracle. The social reading sees Śabarī’s episode as a remarkable act of divine condescension. But the text’s own phenomenology shows something far more radical: nothing happened. Or rather, what happened was the dissolution of the social categories (‘sinful,’ ‘low-caste,’ ‘cursed’) that were never properties of the encountered consciousness but properties of the encountering PF. Rāma did not ‘touch’ Ahalyā despite her being cursed, or ‘accept’ Śabarī’s berries despite her being tribal. The ‘despite’ is the hierarchy. Remove the ‘despite’ and what remains is mutual recognition, Rāma identified himself with them through consciousness, not through social categories or institutional orders. This is the R-operator’s contraction mode (Samanta, 2026b) at its absolute limit: self-magnitude contracting so completely that no position remains from which to perceive social hierarchy.

Third, this connects the structural analysis to the wellbeing framework of non-compensable balance (Samanta, 2026c): liberation is not something added to a person’s existing condition. It is the removal of what was never theirs, the social categories, the commentarial overlays, the hierarchical readings that occluded what was always there. Śabarī’s consciousness was never ‘low.’ Ahalyā was never ‘stone.’ The stone was everyone else’s inability to perceive what was present. The contemplative recognition traditions examined in this article’s companion studies (Samanta, 2026d) converge on this same structural observation: the body knows before the mind names, and what the body knows requires no institutional certification.

## 8 Formal notation

The topological classification can be expressed formally using the Position Fixation (PF) and Liberation Coefficient (LC) framework established in Liberation Mathematics I (Samanta, 2026a). PF measures the degree to which consciousness is fixated in a particular identity-position; LC measures the capacity for perpetual self-transcendence without position-fixation (for full operational definitions, see Samanta, 2026a, Section 2). Let C denote the conditioned principle, U the unconditioned principle, and G the perceived gap. The three topological operations on G are:

Type I (Closed): G → 0, PF → 0, LC → max.

Type II (Open): G > 0 (refined), PF oscillates, LC bounded.

Type III (Disent.): G = natural, PF → 0 (withdrawal), LC exits domain.

The R-operator (Samanta, 2026b) operates within Types I and II but not within Type III, since the R-operator requires relational encounter as its domain condition, and disentanglement resolves through withdrawal from the relational field. These three types exhaust the logical possibilities for a two-principle system: the gap can be eliminated (closed), maintained (open), or recognised as original condition (disentanglement). No fourth operation exists. This claim of topological exhaustion rests on the assumption that the system is binary (two principles with a gap). Systems with more than two principles, or systems that do not model a conditioned–unconditioned distinction (such as the Australian Aboriginal frameworks noted in Section 1.1), fall outside the present analysis (Figure 4).

**Figure 4:**
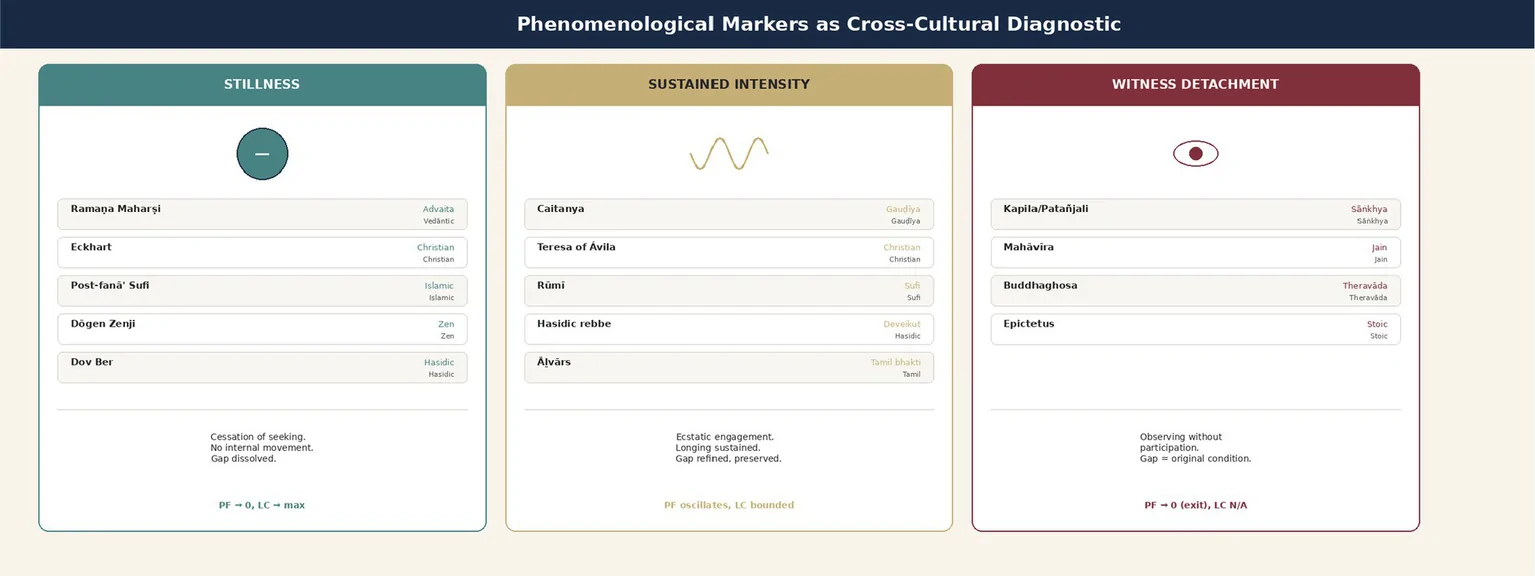
The topological exhaustion argument: three operations exhaust the logical space for a two-principle system.

## 9 Phenomenological markers as cross-cultural diagnostic

Each topological type produces a distinctive phenomenological signature observable across the traditions instantiating it. Stillness (closed): the cessation of existential movement: Ramaṇa Maharṣi’s sahaja sthiti, Eckhart’s Gelassenheit, the post-fanā’ ‘sobriety after intoxication,’ Hasidic prayer where ‘the ego (ani) becomes nought (ayin).’ Sustained intensity (open): ecstatic engagement: Caitanya’s mahābhāva, Teresa’s seventh mansion, Rūmī’s whirling. Witness-detachment (disentanglement): observation without participation: Sāṅkhya’s puruṣa, Theravāda’s bare attention, Stoic apatheia. These markers provide diagnostic value because traditions frequently misclassify their own topology: Eckhart’s relational language (appearing open) masks closed phenomenology; Rūmī’s fanā’ doctrine (appearing closed) masks open phenomenology. A systematic analysis of this mismatch pattern is developed in Article II of this series.

Stillness (closed): the cessation of existential movement. Ramaṇa Maharṣi’s sahaja sthiti, Eckhart’s Gelassenheit, the post-fanā’ ‘sobriety after intoxication,’ Hasidic prayer where ‘the ego (ani) becomes nought (ayin).’ This is not the absence of activity but the absence of seeking. The liberated person may be intensely active but the internal orientation toward an absent goal has ceased, because the goal has been recognised as never having been absent.

Sustained intensity (open): ecstatic engagement rather than cessation. Caitanya’s mahābhāva, Teresa’s seventh mansion, Rūmī’s whirling. The structural distinction from stillness is precise: where closed-topology liberation terminates seeking, open-topology liberation refines seeking into its ultimate form. The relationship between seeker and sought is the final state, not a preliminary to be surpassed.

Witness-detachment (disentanglement): the quietude of a witness who has withdrawn identification from everything that is not pure awareness. Sāṅkhya’s puruṣa, Theravāda’s bare attention, Stoic apatheia. This resembles closed-topology stillness superficially but differs in its internal structure. Closed stillness arises from homecoming (the seeker discovers that what it sought was itself). Disentanglement stillness arises from completed exile (the witness withdraws from everything it is not) (Figure 5).

**Figure 5:**
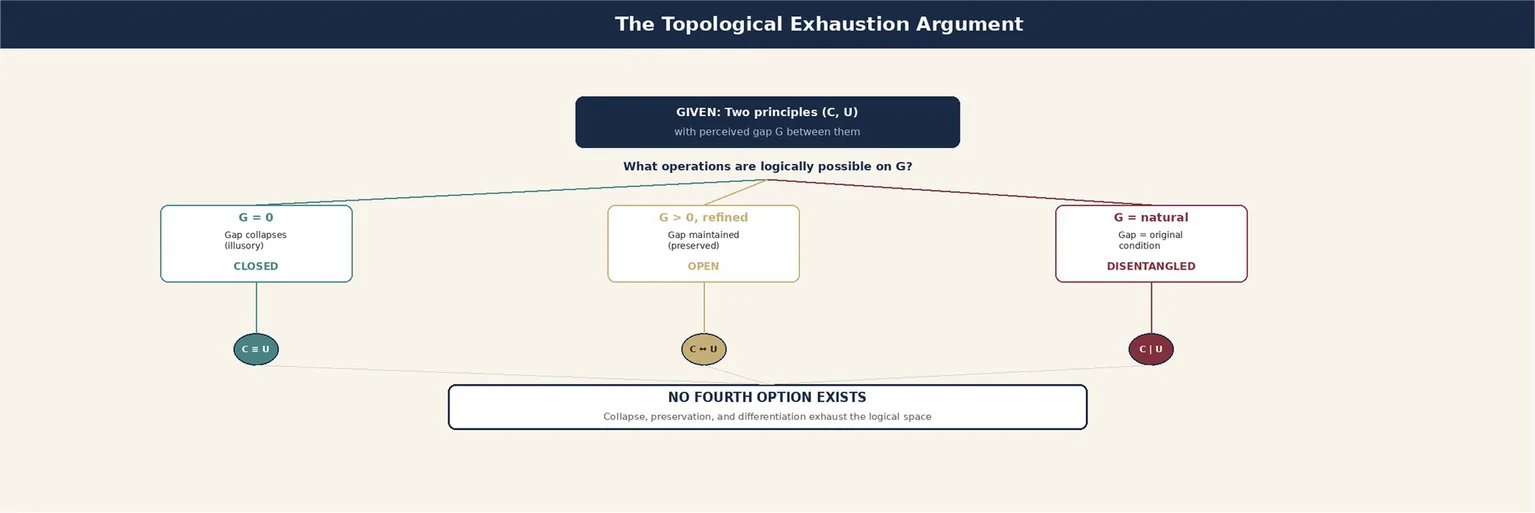
Phenomenological markers as cross-cultural diagnostic: stillness (closed), intensity (open), detachment (disentanglement).

## 10 Limitations

Several significant limitations must be acknowledged. First, the structural-phenomenological method adopted here necessarily abstracts from the lived richness of each tradition. Individual traditions contain multiple and even competing liberation models; the topological classification applies to specific models within traditions, not to traditions as wholes. Sufism alone contains al-Ghazālī (closed), Rūmī (open), and arguably other configurations. The comparative table (Figure 1) should therefore be read as mapping specific models, not entire traditions.

Second, the selection of traditions, while broad, is not exhaustive. African, Indigenous Australian, Mesoamerican, and other traditions warrant examination. As noted in Section 1.1, many of these traditions may not instantiate the two-principle structure that generates liberation topologies, which would itself be a significant finding.

Third, the question of indirect historical influence between traditions complicates the claim of contextual independence in some cases. The article has attempted to distinguish analytical independence (treating traditions as separate comparative cases) from historical independence (demonstrating no contact), and to qualify the stronger claim where the evidence is ambiguous.

Fourth, the assignment of specific traditions to topological types involves interpretive judgment. The criterion (the tradition’s treatment of the gap) is structural, but its application to complex textual corpora is necessarily hermeneutical. A different scholar, emphasising different sub-traditions or texts, might classify some models differently. The framework is falsifiable in the structural sense: the discovery of a liberation model that does not reduce to one of the three operations on G, across traditions operating within a two-principle framework, would constitute a counterexample.

Fifth, the article presents the evidence of convergence but deliberately withholds the causal hypothesis. The companion Article III develops the argument that the convergence reflects structural necessity rather than coincidence or diffusion, but the present article does not make that stronger claim.

Sixth, the formal concepts from Liberation Mathematics (PF, LC, R-operator) are introduced briefly in this article for readers unfamiliar with the companion theoretical papers. Full operational definitions, mathematical formalisations, and empirical validation protocols are available in Samanta (2026a, 2026b). The present article can be read independently of those papers, but the formal notation in Section 8 refers to constructs developed there.

## 11 Conclusion

This article has demonstrated that six civilisational traditions, developing their liberation models within substantially different philosophical and institutional contexts, instantiate a shared five-element structural template that resolves into exactly three topological types: closed, open-relational, and disentanglement. The convergence is structurally precise and spans traditions that differ maximally in doctrinal content, metaphysical commitment, and cultural apparatus.

The Invariance Test introduces a diagnostic criterion drawn from the primary texts themselves: liberation is characterised not by observable transformation but by its absence. The liberated person, Ahalyā returning to her life, Śabarī continuing to taste berries) does not celebrate, advertise, or restructure identity around the event. The social categories through which we read these episodes (‘sinful,’ ‘low-caste,’ ‘cursed’) are not properties of the encountered consciousness but properties of the encountering position-fixation. What the texts describe, when read without the commentarial overlay, is not hierarchical transaction but mutual recognition, consciousness identifying itself with consciousness, without the intermediary of social category.

This convergence demands explanation. The companion articles in this series develop the phenomenological evidence (Article II: phenomenological markers as cross-cultural diagnostics) and the formal hypothesis (Article III: the Universal Liberation Geometry Hypothesis). The vocabulary changes. The geometry remains.

## Data Availability

The original contributions presented in the study are included in the article/supplementary material, further inquiries can be directed to the corresponding author.
